# Preoperative Group and Save Tests in Elective and Emergency Cholecystectomies: An Economic and Environmental Burden

**DOI:** 10.7759/cureus.96294

**Published:** 2025-11-07

**Authors:** Max Moss, Areen Hassan Haleem, Aiman Ibrahim, Camille Kippen, Ian Farrell

**Affiliations:** 1 Trauma and Orthopaedics, Royal Bolton Hospital, Bolton, GBR; 2 General Surgery, Stepping Hill Hospital, Stockport, GBR; 3 General Surgery, Royal Bolton Hospital, Bolton, GBR; 4 General and Colorectal Surgery, Royal Bolton Hospital, Bolton, GBR

**Keywords:** carbon footprint, economic burden of healthcare, laparoscopic cholecystectomy, perioperative blood transfusion, pre operative group and save

## Abstract

Background and objective

Cholecystectomy is among the most frequently performed elective day-case surgeries in the UK, with a growing emphasis on same-day discharge. Although perioperative bleeding and the need for blood transfusion are uncommon with this procedure, routine group and save (G&S) testing is frequently performed for cholecystectomy, contributing to both a significant economic and environmental burden. This study aimed to assess the proportion of patients undergoing cholecystectomy who had preoperative G&S testing and to identify how many required a perioperative blood transfusion.

Methods

This study involved a retrospective analysis of 755 consecutive adult elective or emergency cholecystectomy procedures performed over a two-year period between August 2022 to 2024 in a North West England NHS trust. Patients were assessed from admission through to discharge. Data collected included American Society of Anesthesiologists (ASA) grade, surgical urgency, the number of preoperative G&S tests performed, and the incidence of perioperative red cell transfusion.

Results

Our cohort included 755 patients: 728 (96.4%) underwent total cholecystectomy, with 27 (3.6%) having associated cholecystectomy procedures. Seven hundred (92.7%) patients had an elective procedure, while 55 (7.3%) underwent an emergency cholecystectomy. The mean patient age was 49 years. Of the cohort, 159 patients (21%) were classified as ASA grade 1, 491 (65%) as ASA grade 2, and 105 (14%) as ASA grade 3. A total of 1,422 G&S samples were submitted for this cohort; 481 patients (64%) had two samples taken, while 92 patients (12%) had more than two G&S tests performed. Only one patient required a red cell transfusion, and two patients required a return to theatre due to anaemia secondary to haemorrhage.

Conclusions

Our findings suggest that routine preoperative G&S testing is not required in cholecystectomy, given the paucity of blood product transfusions. Unnecessary G&S testing costs our trust approximately £24,000 and consumes around 2,480 hours of laboratory time. This practice also imposes a considerable financial and environmental burden due to greenhouse gas emissions associated with the testing process. A more selective and stratified approach to G&S testing in cholecystectomy may prove beneficial in economic terms and work in synergy with the goal of improving sustainability in the NHS.

## Introduction

Gallstones affect an estimated 10-15% of the UK population, and surgical management via cholecystectomy represents one of the most commonly performed elective day-case operations globally, accounting for around 60,000 procedures each year in the UK alone [1]. The procedure is associated with low complication rates, allowing a significant number of patients to be eligible for day-case surgery. The proportion of day-case cholecystectomies has risen substantially, from 16% in 2008/09 to 64.8% in 2023 [2].

Perioperative haemorrhage with cholecystectomy is very rare. Retrospective analyses of 14,243 and 22,000 procedures showed that only 2.3% of cases experienced intraoperative bleeding complications, 0.08% had major haemorrhage, and 1.3% required red cell transfusion [3,4]. The risk is similar between laparoscopic and open approaches [5,6]. Nevertheless, clinicians frequently perform preoperative group and save (G&S) testing to determine a patient’s ABO and RhD blood group and to screen for atypical red cell antibodies in case a transfusion becomes necessary.

The clinical value of performing G&S tests for cholecystectomies has come under increasing scrutiny in recent years, with extensive evidence suggesting that it is not a necessary intervention [5,7,8,9,10,11]. In 2016, the National Institute for Health and Care Excellence (NICE) issued guidance on preoperative testing for minor, intermediate, and major surgeries, aiming to clarify which investigations are necessary before elective procedures. However, these guidelines did not include recommendations on G&S testing [12]. In the absence of official guidance, many trusts continue to perform routine G&S tests before cholecystectomies, potentially influenced by local policies, clinical caution, or habitual practice [11]. This continues despite evidence that discontinuing routine testing could save both time and money without compromising patient safety.

There is a growing recognition of the environmental impact of healthcare practices, which are responsible for approximately 5.2% of global greenhouse gas emissions and a large proportion of the UK domestic carbon footprint through the clinical consumable items [13,14]. While G&S testing accounts for only a small fraction of these emissions, limiting its use when not clinically necessary could help reduce the environmental burden.

At our trust, the decision to order preoperative G&S testing before cholecystectomy is based on independent clinical judgement, as there is no formal local guidance. This study aimed to evaluate the number of patients undergoing cholecystectomy who received preoperative G&S testing and the number who required perioperative transfusion, allowing comparison of our findings with existing research. Secondary outcomes included analysis of the cost and environmental burden associated with current practice. These findings may stimulate further discussion regarding the necessity of G&S testing in elective cholecystectomy and prompt reflection on the sustainability of such interventions.

## Materials and methods

Study design and setting

This was a retrospective cohort study of 755 consecutive adult patients who underwent either laparoscopic or open cholecystectomy at Royal Bolton Hospital, UK, and their preoperative G&S testing practices between August 2022 and August 2024. Since this involved a single-centre service evaluation and audit, formal ethical approval was not required for this in line with internal governance guidelines. The study sought to evaluate current clinical practice within the trust in relation to existing research on cholecystectomies and transfusions, to help contribute to the discussion around the economic and environmental impact of these practices.

Inclusion and exclusion criteria

Inclusion criteria consisted of patients aged 16 years or older undergoing elective or emergency laparoscopic or open cholecystectomy. Patients were identified using the following ICD-10 (the 10th revision of the International Classification of Diseases) codes: J18.1 (total cholecystectomy with excision of surrounding tissue), J18.3 (other specified total cholecystectomy), J18.4 (partial cholecystectomy with exploration of the common bile duct (CBD), J18.5 (other specified partial cholecystectomy), J18.8 (other specified excision of the gallbladder), and J18.9 (unspecified excision of the gallbladder). Patients were excluded if they were <16 years old or if inadequate documentation prevented accurate data collection.

Outcomes

The primary outcome was the number of G&S tests performed per patient undergoing cholecystectomy. Secondary outcomes included the number of perioperative red blood cell transfusions and the number of cases that required return to theatre.

Data collection and analysis

Data were collected retrospectively from the electronic hospital records system ‘ICE’ (Integrated Clinical Environment) and included patient demographics (age, sex), American Society of Anesthesiologists (ASA) grade, and the number of G&S samples submitted per patient (during admission and at preoperative outpatient clinics when elective cholecystectomy was listed). This system allowed the review of transfusion records from the date of surgery through discharge to identify any instances of transfusions. The number of transfusions was described as a percentage of patients within the sample who received a perioperative transfusion. Data were collected and analysed descriptively using Microsoft Excel, Version 16.101.3 (Microsoft Corporation, Redmond, WA).

## Results

Of the 755 patients included in the study, 728 (96.4%) underwent total cholecystectomy. Among the remaining patients, 21 (2.8%) had partial cholecystectomy, two (0.3%) underwent total cholecystectomy with excision of surrounding tissue, three (0.4%) had total cholecystectomy with CBD exploration, and one (0.1%) patient underwent an unspecified excision of the gallbladder (Table 1).

**Table 1:** Distribution of surgical procedures CBD: common bile duct

Procedure	N (%)
Total cholecystectomy	728 (96.4%)
Partial cholecystectomy	21 (2.8%)
Total cholecystectomy and excision of surrounding tissue	2 (0.3%)
Total cholecystectomy and CBD exploration	3 (0.4%)
Unspecified gallbladder excision	1 (0.1%)

The average age of the patients included in the study was 49 years (range: 19-87 years). Among them, 491 patients (65%) had an ASA grade of 2, 159 (21%) had an ASA grade of 1, and 105 (14%) were classified as ASA grade 3 (Table 2). Of the total 755 patients, 700 (93%) underwent elective cholecystectomy, while the remaining 55 patients (7%) underwent emergency procedures (Table 3).

**Table 2:** Distribution of demographics and ASA grades ASA: the American Society of Anesthesiologists

ASA grade	N (%)
ASA 1	159 (21%)
ASA 2	491 (65%)
ASA 3	105 (14%)

**Table 3:** Distribution of elective and emergency cholecystectomies

Type of surgery	N (%)
Elective	700 (93%)
Emergency	55 (7%)

In total, 1,422 G&S samples were submitted for these patients. A total of 481 (64%) patients had two G&S samples submitted, in line with the standard protocol. However, 92 patients (12%) had more than two samples sent, with the maximum being eight samples for a single patient. Analysis of the results revealed that a total of 55 G&S samples were rejected by the laboratory (Table 4). Two (0.3%) patients in this cohort returned to surgery for further procedures. Recorded administration of blood products was only found in one of these patients.

**Table 4:** Number of G&S samples sent per patient and the number of G&S samples rejected G&S: group and save

Category	N (%)
Total G&S samples submitted	1,422
Patients with two G&S samples	481 (64% of patients)
Patients with more than two samples	92 (12% of patients)
Rejected G&S samples	55

The first case was a 19-year-old female patient (ASA grade 1) who, three days post-laparoscopic cholecystectomy, was found to have a low haemoglobin level and received two units of red blood cell transfusion. She was subsequently taken back to the theatre, where a slow bleed from the liver bed was identified. This was the only case where the G&S samples were utilised. The second case involved a 41-year-old male patient (ASA grade 3) who experienced a haemoglobin drop one day after surgery. He was initially managed conservatively but was taken back to the theatre on postoperative day seven for a repeat laparoscopy. A four-quadrant haemoperitoneum was found, although no specific bleeding source was identified. This patient did not require a transfusion. Both patients made a full recovery. Neither had any identifiable predisposing factors that would have increased their risk of postoperative bleeding. 

## Discussion

Analysis of our data demonstrated a perioperative transfusion rate of 0.1% following cholecystectomy, indicating that this is a rare complication and aligning with findings from previous studies. One previous retrospective review of 562 laparoscopic procedures over two years reported a transfusion rate of 0.18% [15]. Furthermore, two earlier studies demonstrated a 0.9% transfusion rate in laparoscopic cholecystectomies, with another at 0.46% [10,16]. Larger systematic reviews examining over 40,000 patients also demonstrated a transfusion rate of 0.1% for laparoscopic and a bleeding complication rate of 0.4% for open cholecystectomies, reinforcing the rarity of significant bleeding complications [5,6]. These findings, together with our results, support making G&S testing non-mandatory for cholecystectomies. They also highlight the need for clearer national guidelines to establish a standardised framework and reduce variability in clinical practice for these procedures.

One case within our cohort required a blood transfusion, which was administered three days postoperatively. There was sufficient time to obtain and process two G&S samples and crossmatch blood for patient administration; similar findings and practices have been reported in other cohort studies. [7]. No cases of major intraoperative bleeding occurred in our cohort, consistent with previous studies on major intraoperative haemorrhage in cholecystectomy: 0.1% and 2.3% in nearly 10,000 cases in two separate studies [17,18]. In the rare event of major vascular injury, delaying transfusion to obtain crossmatched blood may pose significant risks to patient safety. Even when two valid G&S samples are available, immediate transfusion with uncrossmatched group O blood is often required in major haemorrhage [2,12]. Several studies have suggested that O-negative blood should be readily available in the theatre to cover such emergencies [10,19,20].

Laboratory pathology investigations cost the NHS approximately £2.5 billion annually in the UK [21], with the Carter Review identifying a large volume of pathology testing as unnecessary. G&S testing costs vary - one study of 4,462 patients who underwent cholecystectomy estimated the cost of a G&S sample to be £20 (excluding VAT), with approximately 120 minutes of laboratory processing time per sample [22]. Another provided a similar estimated cost of £17.24 per sample [10]. Based on these figures, the 1,420 unused G&S samples in our study represent an estimated financial loss of approximately £24,481 and a waste of 2,840 hours of laboratory time over the two-year study period. These figures exclude the additional costs of consumables such as blood tubes and needles, and do not account for collection, transport, or administrative processing [10]. When extrapolated to the approximately 60,000 cholecystectomies performed annually across the UK [1], discontinuing routine preoperative G&S testing could yield savings exceeding £1 million per year, a significant cost and a viable way to reduce costs in a resource-constrained system like the NHS.

The Lancet’s 2022 report on climate and health estimated that the global healthcare sector accounts for roughly 5.2% of total greenhouse gas emissions, with the NHS representing a major contributor to the UK’s overall carbon footprint [13,14]. Further studies from The Lancet in 2019 show the NHS produced 25 megatonnes of CO₂e (equivalent) [23]. Although there has been an improvement in the sustainability of the NHS, additional initiatives are needed to build on this success. Although a single G&S test has a minimal individual impact, the cumulative effect of unnecessary testing across thousands of cases becomes significant. Each test requires single-use plastics, chemical reagents, and energy-intensive equipment, all of which generate emissions, with earlier-mentioned studies estimating approximately 82 g CO₂e per haematological test [13].

A 2024 NHS single-trust study examining 15,293 operations and 637 red cell requests calculated the carbon footprint of a G&S test at 0.43 kg CO₂e per inpatient and 7 kg CO₂e per outpatient [24]. In our cohort, 1,422 G&S samples were submitted, but only two were used. The 1,420 unused samples are estimated to have produced 610 kg CO₂e, equivalent to more than two flights from London to Paris, charging a smartphone 143,000 times, 240 kg of incinerated plastic, or nearly 600,000 recycled aluminium cans [25]. Further extrapolating these numbers to nationwide cholecystectomies, unnecessary preoperative G&S testing will have a marked annual contribution to greenhouse gas emissions. This stands in contrast to the goal of greener surgical practice within the NHS. Given the low transfusion rate in cholecystectomies (<1%), discontinuing routine G&S testing in these cases could significantly reduce environmental waste.

Limitations

This study has several limitations. Its retrospective design inherently limits the accuracy and completeness of data, as it relies on the quality and consistency of electronic health records. Secondly, the study was conducted within a single NHS trust, which may limit the generalisability of the findings to other institutions due to the small sample size. This also predisposes towards a selection bias with surgeons testing patients based on their independent experience of 'high-risk patients' without the use of evidence-based guidance. Additionally, the study only captured transfusion events occurring during the index hospital admission, thereby excluding any delayed postoperative bleeding or transfusions that may have occurred after discharge, potentially underestimating the true incidence.

Furthermore, human error likely contributed to unnecessary testing and inefficiency, with instances of multiple G&S samples being taken despite valid samples already existing, possibly due to short-notice procedure cancellation, inadequate communication, or lack of awareness of previous results. This may also have contributed to the rejection of 55 samples, possibly due to labelling mistakes, improper sample handling, or timing issues related to collection protocols. These factors introduce variability and may have inflated the number of samples submitted, thereby affecting the accuracy of the environmental and financial burden calculations. Finally, this cohort included procedures outside of routine cholecystectomy, such as biliary exploration, that run a higher risk of vascular damage and prompt surgeons to prepare for transfusion; this may also limit the generalisability of our data to higher risk cases.

## Conclusions

This study shows that perioperative blood transfusion following elective and emergency cholecystectomy is exceedingly rare. Despite the low clinical utility, over 1,400 G&S samples were processed, the vast majority of which were unused. This practice contributes to significant financial waste, unnecessary use of laboratory resources, and a measurable environmental impact. We recommend additional multi-centre studies to inform policy-level recommendations, while recognizing the inherent limitations of a single-centre analysis. Climate change threatens to jeopardise the healthcare system’s ability to deliver high-quality care. In light of these findings and the lack of clear national guidance, there is a strong case to revise current preoperative protocols by removing routine G&S testing for low-risk procedures such as elective cholecystectomy. This change would promote a more sustainable, cost-effective, and clinically sound healthcare system.
